# Cognitive transparency and trust calibration in explainable AI systems: a psychological model of human-AI decision reliance

**DOI:** 10.3389/fpsyg.2026.1924913

**Published:** 2026-10-14

**Authors:** Usman Rehman

**Affiliations:** Department of Global Venture Technology Management, Hoseo University, Seoul, Republic of Korea

**Keywords:** explainable artificial intelligence (XAI), cognitive transparency, trust calibration, human-AI interaction, algorithmic decision-making, AI trust, decision reliance, AI literacy

## Abstract

**Introduction:**

Explainable artificial intelligence (XAI) is widely expected to improve transparency and support appropriate human reliance on AI-assisted decision-making. However, the psychological processes through which explainability relates to reliance remain insufficiently understood, and explainability, transparency, and trust have largely been examined separately.

**Objectives:**

This study develops and tests a psychological model of cognitive transparency in which AI explainability, algorithmic transparency, and perceived AI accuracy are associated with human reliance on AI through cognitive understanding, cognitive trust, and trust calibration, with AI literacy as a boundary condition.

**Methods:**

A cross-sectional online survey was completed by 342 AI users in South Korea in May and June 2026. Each participant evaluated one self-selected AI system that they had personally used; all constructs were measured by self-report. The data were analyzed using partial least squares structural equation modeling (PLS-SEM) in SmartPLS 4.

**Results:**

AI explainability (β = 0.349) and algorithmic transparency (β = 0.297) were positively associated with cognitive understanding. Cognitive understanding (β = 0.388) and, more strongly, perceived AI accuracy (β = 0.492) were associated with cognitive trust. Cognitive trust was associated with trust calibration (β = 0.631), which was associated with human reliance on AI (β = 0.600). All hypothesized indirect effects were significant, and AI literacy strengthened the association between AI explainability and cognitive understanding (β = 0.192). The model explained 36%–54% of the variance in the endogenous constructs and showed medium predictive power.

**Conclusion:**

The findings are consistent with a sequence in which explainable AI relates to reliance through users’ understanding and trust judgments. Because all constructs were self-reported and measured at a single point in time, the results describe associations among users’ perceptions rather than causal effects, independently verified calibration, or objectively appropriate reliance. The findings suggest that AI developers in domains such as healthcare, education, and finance, where calibrated reliance is critical, should pair user-facing explanations with information about system accuracy and with AI literacy support.

## 1 Introduction

Artificial intelligence (AI) systems increasingly support how individuals acquire information, solve problems, and make decisions. Recommendation engines, predictive analytics platforms, intelligent assistants, and generative AI models are now widely used in education, healthcare, finance, business, and public administration (Kasneci et al., 2023; Dwivedi et al., 2023; Rehman, 2026a). As these systems take on a larger role in supporting human judgment, understanding the psychological factors associated with people’s trust in and reliance on AI has become an important research priority.

A persistent obstacle is that many advanced AI models, particularly deep learning systems, generate outputs without revealing how those outputs were reached, leaving users uncertain about whether and when to rely on them (Gunning and Aha, 2019; Miller, 2019; Buçinca et al., 2021; Lai et al., 2023). Explainable artificial intelligence (XAI) seeks to address this opacity by providing interpretable explanations of how AI systems generate predictions and recommendations (Ribeiro et al., 2016; Adadi and Berrada, 2018). Explanations are expected to help users evaluate AI outputs and interact with AI systems in a more informed way (Miller, 2019; Shin, 2021), and empirical work suggests that understandable AI support is associated with greater engagement and satisfaction, including in AI-assisted learning environments (Rehman, 2026b).

From a psychological perspective, trust in AI is not determined solely by objective system performance. Rather, it rests on users’ evaluations of system characteristics such as competence, reliability, and predictability (Mayer et al., 1995; McKnight et al., 2002; Hoff and Bashir, 2015; Glikson and Woolley, 2020; Rehman, 2026a). Trust in automation research further emphasizes that the goal is not maximal trust but calibrated trust, that is, trust that corresponds to a system’s actual capabilities (Lee and See, 2004). Excessive trust can lead to misuse of automation, whereas insufficient trust can lead to disuse of valuable support (Parasuraman and Riley, 1997). Explanations are often proposed as a means of supporting such calibration (Buçinca et al., 2021; Lai et al., 2023; Mehrotra et al., 2024), and recent reviews identify appropriate trust as a central requirement for effective human-AI collaboration (Bach et al., 2024; Mehrotra et al., 2024).

Despite this growing literature, three gaps remain. First, explainability, algorithmic transparency, perceived accuracy, and trust have mostly been examined as separate antecedents of AI acceptance or behavioral intention (Shin, 2021; Dwivedi et al., 2023). Relatively few studies have examined, within a single model, how users’ perceptions of explainable AI relate to reliance through their understanding of the system, their trust in it, and their judgment of whether that trust is appropriately calibrated. Second, perceived accuracy, a well-established determinant of trust in automation (Hoff and Bashir, 2015; Glikson and Woolley, 2020), is rarely considered together with explainability and transparency, even though users are likely to evaluate these system characteristics jointly (Rehman, 2026a,c). Third, although AI literacy is increasingly recognized as a capability that shapes how people interpret AI outputs (Long and Magerko, 2020; UNESCO, 2024), few studies have examined whether it conditions the association between explainability and users’ understanding of AI systems.

To address these gaps, the present study develops and tests a model of *cognitive transparency* in explainable AI systems, drawing on XAI research, trust theory, and trust in automation theory. Cognitive transparency refers to the user-side counterpart of technical explainability: the extent to which users experience an AI system’s reasoning as understandable (section “2.4 Cognitive transparency and related constructs”). The model proposes that users’ perceptions of AI explainability and algorithmic transparency are associated with their cognitive understanding of an AI system; that cognitive understanding and perceived AI accuracy are associated with cognitive trust; that cognitive trust is associated with trust calibration; and that trust calibration is associated with human reliance on AI-assisted decision-making. AI literacy is examined as a moderator of the association between AI explainability and cognitive understanding.

The scope of this study should be stated at the outset. All constructs were measured through self-report in a cross-sectional survey in which participants evaluated an AI system of their own choosing. The study therefore tests whether the pattern of associations among users’ perceptions is consistent with the theoretically proposed sequence. It does not establish causal direction, capture how trust develops over time, or assess whether participants’ trust is objectively calibrated or their reliance objectively appropriate.

Within this scope, the study makes four contributions. First, it integrates XAI, trust theory, and trust in automation theory into a single model and tests the sequential indirect associations implied by these perspectives, rather than examining explainability, trust, or acceptance in isolation. Second, it examines perceived accuracy alongside AI explainability and transparency, showing their relative associations with understanding and trust. Third, it introduces cognitive transparency as a user-centered framing of explainability, distinguishing users’ experience of understandable AI reasoning from the technical disclosure of model operations. Fourth, it tests AI literacy as a boundary condition of the association between AI explainability and cognitive understanding. The findings also offer practical guidance for designing AI systems and AI literacy initiatives that support informed reliance. Accordingly, the study addresses the following research questions:

*RQ1: How is cognitive transparency in explainable AI systems, reflected in users’ perceptions of AI explainability and algorithmic transparency and in their cognitive understanding, related to cognitive trust, trust calibration, and human reliance on AI-assisted decision-making?*

*RQ2: Does AI literacy strengthen the association between AI explainability and cognitive understanding?*

## 2 Theoretical background

### 2.1 Trust theory

Trust theory provides a foundation for understanding how individuals come to trust technologies. According to Mayer et al. (1995), trust rests on evaluations of a trustee’s ability, integrity, and benevolence. A distinction is commonly drawn between cognitive trust, which rests on rational evaluations of a trustee’s competence, reliability, and dependability, and affective trust, which arises from emotional bonds and perceived care (McKnight et al., 2002; Glikson and Woolley, 2020). Because the present study concerns users’ judgments of AI systems as decision aids, it focuses on cognitive trust; affective trust is not examined.

Research on trust in AI conceptualizes trust as something that is updated as users observe system behavior over repeated interactions (Glikson and Woolley, 2020; Lai et al., 2023; Rehman, 2026a). This dynamic view is a theoretical premise of the literature on which the present model draws. The present cross-sectional study, however, measures trust at a single point in time and cannot examine how trust changes.

### 2.2 Trust in automation and trust calibration

Trust in automation theory extends trust concepts to human–machine interaction. Lee and See (2004) define trust in automation as the attitude that an automated agent will help an individual achieve goals in situations characterized by uncertainty and vulnerability. A central concept in this theory is trust calibration: the correspondence between a person’s trust and the system’s actual capabilities. Overtrust can lead to misuse, in which users accept erroneous recommendations, whereas distrust can lead to disuse, in which valuable assistance is rejected (Parasuraman and Riley, 1997; Lee and See, 2004; Hoff and Bashir, 2015). Recent experimental evidence shows that such overreliance remains common: users frequently follow AI advice even when it contradicts other available information or their own judgment (Klingbeil et al., 2024).

Two features of this concept are important for the present study. First, calibration is defined relative to system capability, so assessing it directly requires comparing users’ trust with independent evidence of system performance. Second, calibration is conceptually distinct from reliance: calibration concerns the fit between trust and system capability, whereas reliance is the behavioral act of depending on the system, and poorly calibrated trust can also produce high reliance (Parasuraman and Riley, 1997; Buçinca et al., 2021). Because the present study did not measure system performance, trust calibration is operationalized here as users’ *self-assessed* calibration: their judgment that their trust matches the system’s performance and that they can judge when to trust it. Throughout this article, the term trust calibration therefore refers to this perceived calibration, which is a metacognitive judgment and may diverge from actual calibration.

### 2.3 Explainable artificial intelligence

Explainable artificial intelligence aims to make AI decision processes understandable and interpretable to users (Ribeiro et al., 2016; Adadi and Berrada, 2018; Gunning and Aha, 2019; Barredo Arrieta et al., 2020). Beyond their technical functions, explanations are thought to serve psychological functions: they can reduce uncertainty, support users’ reasoning, and shape perceptions of fairness and accountability (Ehsan et al., 2021; Lai et al., 2023). A central mechanism proposed in this literature is that explanations help users form mental models of how a system operates, enabling them to anticipate its behavior and evaluate its outputs (Bansal et al., 2019; Kelly et al., 2023). However, explanations do not always improve decisions. Their benefit depends largely on whether they allow users to verify the correctness of an AI output (Fok and Weld, 2024), and explanations can increase users’ confidence in AI outputs without improving the accuracy of their judgments (Steyvers et al., 2025). In the present study, explainability and algorithmic transparency are examined as users’ perceptions of their chosen system, not as technical properties of that system.

### 2.4 Cognitive transparency and related constructs

Several concepts in this literature are closely related, so their meanings and their relation to the measured constructs are clarified here.

*Cognitive transparency* is the organizing concept of this study. Whereas algorithmic transparency concerns whether a system’s processes are visible, cognitive transparency concerns whether users experience the system’s reasoning as understandable: it is the psychological outcome that explainable AI aims to achieve on the user side. Cognitive transparency is used here as a theoretical framing concept rather than as a separate latent variable. It is not measured with its own items; instead, it is represented empirically by the first segment of the research model, namely the associations of AI explainability and algorithmic transparency with cognitive understanding (H1 and H2) and the conditioning role of AI literacy (H7). The research question therefore asks how this cognitive transparency segment relates to the trust-related constructs that follow it, rather than treating cognitive transparency and its components as distinct variables.

*Mental models* are users’ internal representations of how a system operates (Bansal et al., 2019; Kelly et al., 2023). Mental models were not measured in this study. They serve as the theoretical mechanism explaining why explainability should be associated with understanding.

Cognitive understanding is a system-specific, self-reported judgment that one comprehends how a particular AI system works, can interpret its outputs, and grasps its decision process. It can be regarded as a user’s subjective sense of having a coherent mental model of the system, and it is the measured construct closest to the idea of cognitive transparency. It is not a measure of demonstrated comprehension, and users may overestimate their understanding.

*AI literacy* is a general, person-level capability: knowledge of and familiarity with AI technologies in general, independent of any specific system (Long and Magerko, 2020; Pinski and Benlian, 2024). AI literacy and cognitive understanding therefore differ in their target (AI in general versus one particular system) and in their stability (a relatively enduring individual characteristic versus a judgment about a specific system). AI literacy is likewise distinct from cognitive transparency: cognitive transparency describes a user’s experience of a specific system’s reasoning, whereas AI literacy is a capability the user brings to any system. This distinction is what allows AI literacy to function as a boundary condition: general knowledge of AI may help users translate a particular system’s explanations into system-specific understanding.

### 2.5 Integration of theoretical perspectives

The three perspectives are combined in a single model. XAI research suggests that AI explainability and transparency are associated with users’ cognitive understanding. Trust theory suggests that cognitive understanding and perceived accuracy are associated with cognitive trust. Trust in automation theory suggests that cognitive trust is associated with users’ judgments about the calibration of their trust, which in turn are associated with reliance. Unlike technology acceptance frameworks that focus on behavioral intention (Davis, 1989; Venkatesh et al., 2003), this model focuses on the understanding- and trust-related judgments proposed to link perceptions of explainable AI to reliance. The sequence is a theoretical ordering; the present study tests the associations it implies using cross-sectional data.

## 3 Hypotheses development and research model

### 3.1 Explainability and transparency → cognitive understanding

Explainable artificial intelligence aims to improve users’ understanding of AI systems by providing clear, interpretable explanations of how recommendations are generated (Ribeiro et al., 2016; Adadi and Berrada, 2018). Algorithmic transparency refers to the extent to which a system’s processes, logic, and decision criteria are visible to users (Shin, 2021). When users perceive that a system explains its outputs and makes its decision process visible, they have more information from which to construct a mental model of the system (Bansal et al., 2019; Kelly et al., 2023), and they should therefore be more likely to report that they understand how it works and can interpret its outputs (Shin, 2021; Rehman, 2026a). Accordingly:

*H1: AI explainability is positively associated with cognitive understanding.*

*H2: Algorithmic transparency is positively associated with cognitive understanding.*

### 3.2 Cognitive understanding and cognitive trust

Trust theory holds that trust develops through evaluations of a trustee’s competence, reliability, and predictability (Mayer et al., 1995). Users who believe they understand how an AI system produces its recommendations are better placed to judge its strengths and limitations and to anticipate its behavior, which should support confidence in the system (Shin, 2021; Kelly et al., 2023). Conversely, a lack of understanding leaves users uncertain about the basis of the system’s outputs (McKnight et al., 2002; Hoff and Bashir, 2015). Accordingly:

*H3: Cognitive understanding is positively associated with cognitive trust.*

### 3.3 Perceived AI accuracy and cognitive trust

Perceived AI accuracy refers to users’ evaluation that a system consistently produces correct and dependable outputs. Because users typically lack direct access to objective performance data, their trust depends largely on such perceptions (Hoff and Bashir, 2015). Consistent with the ability dimension of trust (Mayer et al., 1995), users who perceive a system as accurate should regard it as more competent and therefore trust it more (Glikson and Woolley, 2020; Rehman, 2026a,c). Accordingly:

*H4: Perceived AI accuracy is positively associated with cognitive trust.*

### 3.4 Cognitive trust and trust calibration

Trust in automation theory holds that users regulate their trust in light of their evaluations of system performance (Lee and See, 2004). Users who have formed a considered judgment that a system is competent and reliable have an explicit reference point against which to assess whether their trust fits the system’s performance, and they may therefore feel more able to judge when the system can be trusted (Glikson and Woolley, 2020; Bansal et al., 2021). At the same time, high trust can coexist with poor calibration, as in cases of overtrust (Parasuraman and Riley, 1997). This hypothesis therefore concerns users’ perceived, not actual, calibration:

*H5: Cognitive trust is positively associated with trust calibration.*

### 3.5 Trust calibration and human reliance on AI

As noted in section “2.2 Trust in automation and trust calibration,” calibration concerns the correspondence between trust and system capability rather than reliance itself, and poorly calibrated trust can also lead to high reliance through automation bias or overreliance (Parasuraman and Riley, 1997; Buçinca et al., 2021). The argument advanced here is therefore narrower. Users who believe that their trust matches the system’s performance and who feel able to judge when the system can be trusted have a subjective basis for acting on its recommendations. This reduces the uncertainty that otherwise discourages the use of AI advice (Lee and See, 2004; Hoff and Bashir, 2015). Perceived calibration is therefore expected to be positively associated with human reliance. This association does not indicate whether reliance is objectively appropriate:

*H6: Trust calibration is positively associated with human reliance on AI.*

### 3.6 The moderating role of AI literacy

The value of an explanation depends not only on its content but also on the recipient’s capacity to interpret it. Users with greater general knowledge of AI technologies possess existing knowledge structures, such as familiarity with how AI systems learn from data and generate outputs, into which a particular system’s explanations can be integrated (Long and Magerko, 2020; Kasneci et al., 2023). For such users, perceiving that a system provides explanations should translate more readily into system-specific understanding. Users with limited AI literacy may find even well-designed explanations difficult to interpret (Shin, 2021). Because AI literacy is defined as general knowledge of AI and cognitive understanding as a judgment about a specific system (section “2.4 Cognitive transparency and related constructs”), this hypothesis concerns the interaction between a general capability and a system-specific perception rather than a relationship between equivalent constructs:

*H7: AI literacy positively moderates the association between AI explainability and cognitive understanding, such that the association is stronger among users with higher AI literacy.*

### 3.7 Indirect effects

Taken together, the preceding arguments imply that AI explainability is related to reliance not directly but through a sequence of intervening judgments. AI explainability is expected to relate to cognitive trust through cognitive understanding; cognitive understanding is expected to relate to trust calibration through cognitive trust; and cognitive trust is expected to relate to human reliance through trust calibration. Because the data are cross-sectional, these indirect effects test whether the associations are statistically consistent with the proposed sequence rather than establishing causal mediation:

*H8: Cognitive understanding mediates the association between AI explainability and cognitive trust.*

*H9: Cognitive trust mediates the association between cognitive understanding and trust calibration.*

*H10: Trust calibration mediates the association between cognitive trust and human reliance on AI.*

### 3.8 Research model

Figure 1 presents the research model. In line with recommended practice for moderation analysis, the main effect of AI literacy on cognitive understanding was also estimated (Hair et al., 2022).

**FIGURE 1:**
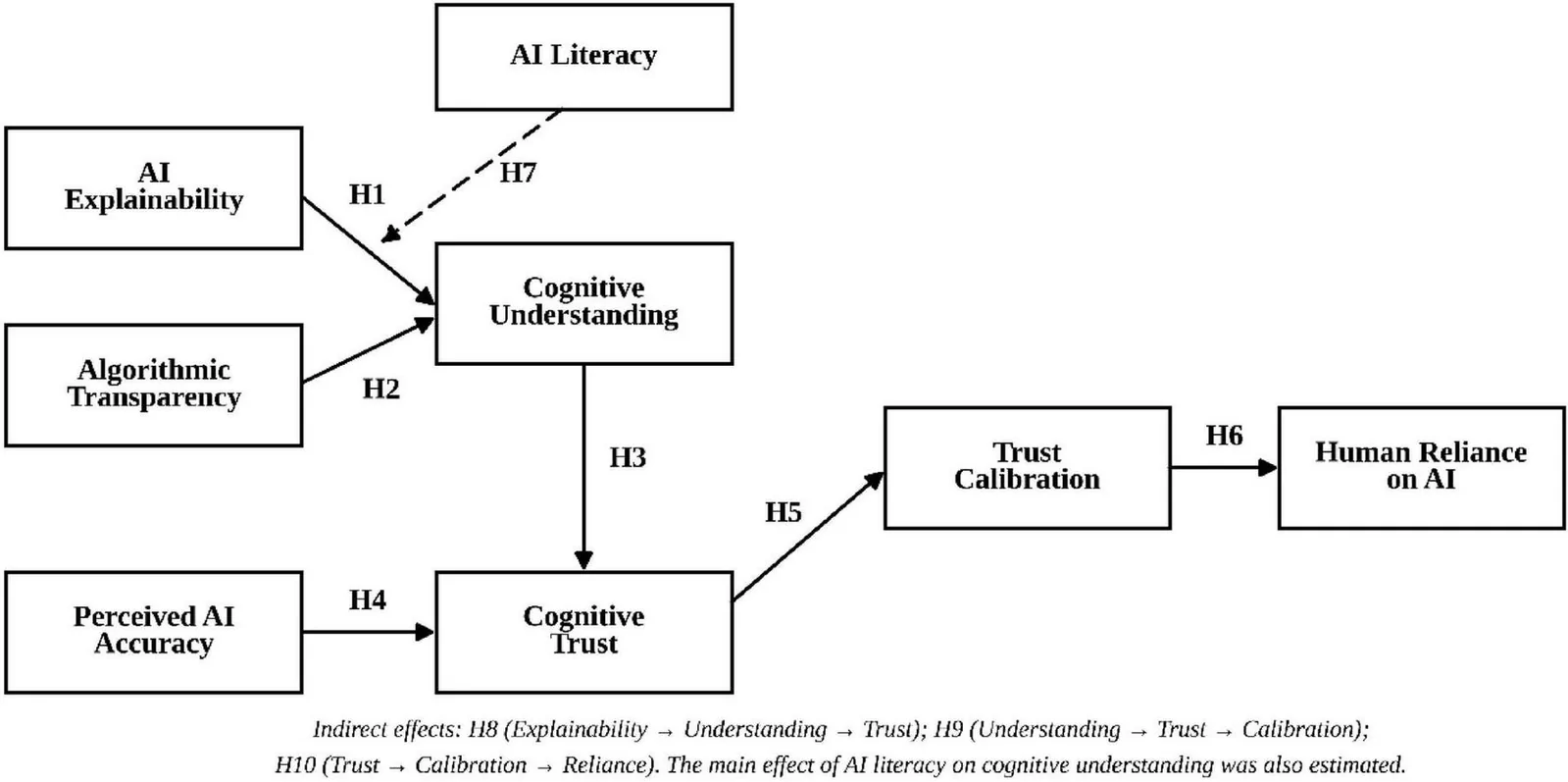
Research model. Solid arrows indicate hypothesized direct associations (H1–H6); the dashed arrow indicates the hypothesized moderating effect of AI literacy (H7). All constructs were measured through self-report.

## 4 Materials and methods

### 4.1 Research design

This study used a quantitative, cross-sectional survey design to test the associations proposed in the research model. Partial least squares structural equation modeling (PLS-SEM) was used because the model contains multiple latent constructs as well as mediating and moderating relationships, and because the study has a prediction-oriented purpose (Hair et al., 2022). A cross-sectional design allows the associations among constructs to be tested but does not allow causal or temporal ordering to be established.

### 4.2 Participants and procedure

The study was conducted in South Korea, where AI technologies are widely used in education, business, and public services. The target population comprised university students and early-career professionals with prior experience using AI-based systems. Participants were recruited through a non-probability convenience sampling approach via universities, academic networks, and professional communities, and completed an online questionnaire. Inclusion criteria were being at least 18 years of age, having prior experience using AI-based systems, and agreeing voluntarily to participate. Data were collected in May and June 2026.

At the beginning of the questionnaire, participants were informed of the academic purpose of the study, the voluntary and anonymous nature of participation, and their right to withdraw at any time. No personally identifiable information was collected. Participants then received the following instructions (see also Supplementary Section 1): “Please think about an AI-based system or application that you have personally used and are familiar with. This may include generative AI platforms, AI-powered recommendation systems, intelligent assistants, predictive analytics applications, or other AI-supported decision-making systems. For all questions that follow, please keep the same AI system in mind and answer based on your actual experience with that system. Please indicate the response that best reflects your perceptions, experiences, and interactions with the AI system. There are no right or wrong answers.” All items referring to “the AI system” therefore reflect participants’ perceptions of one self-selected system. The type of system each participant chose was not recorded.

A total of 463 responses were received. Responses were screened for completeness and duplication: incomplete questionnaires (i.e., responses with one or more unanswered items on the study scales; *n* = 93) and duplicate submissions (*n* = 28) were excluded, resulting in the removal of 121 responses (26.1%). The final sample comprised 342 valid responses (usable response rate = 73.9%), with no missing values. The questionnaire collected gender, age group, and frequency of AI use; educational and occupational characteristics were not collected. The sample size exceeds commonly recommended minimum requirements for PLS-SEM models of this complexity (Hair et al., 2022).

### 4.3 Measures

Because established multi-item measures for several constructs in the AI-assisted decision-making context were not available, the measurement items were developed for this study based on the conceptual and empirical literature on each construct: explainable AI (Ribeiro et al., 2016; Adadi and Berrada, 2018; Miller, 2019), algorithmic transparency (Shin, 2021), perceived accuracy and reliance on automation (Hoff and Bashir, 2015), trust (Mayer et al., 1995; McKnight et al., 2002), trust calibration (Lee and See, 2004), and AI literacy (Long and Magerko, 2020). Items were worded to refer to the participant’s self-selected AI system, except for the AI literacy items, which refer to AI technologies in general. Each of the eight constructs was measured with three reflective items. Accordingly, cognitive understanding, trust calibration, and human reliance on AI reflect participants’ self-reports rather than demonstrated comprehension, independently assessed calibration, or observed reliance behavior. No formal pilot test or expert panel review was conducted before data collection; evidence of reliability and validity therefore derives from the measurement model assessment in the present sample (section “5.2 Measurement model”).

The questionnaire was administered in English. Responses were recorded on a five-point Likert scale ranging from 1 (strongly disagree) to 5 (strongly agree). No items were reverse-coded. The item responses were entered as reflective indicators in SmartPLS 4, which estimated construct scores as weighted composites of their indicators. The complete survey instructions and all administered items, together with their conceptual basis, item codes, means, and standard deviations, are reported in Supplementary Tables 1, 2.

### 4.4 Data analysis

The data were analyzed with SmartPLS 4 (version 4.1.1.4; Ringle et al., 2024) using the path weighting scheme, a maximum of 3,000 iterations, and a stop criterion of 10^–7^. The measurement model was assessed through indicator loadings, internal consistency reliability (Cronbach’s α, ρA, and composite reliability ρC), convergent validity (average variance extracted, AVE), and discriminant validity [the Fornell-Larcker criterion, the heterotrait-monotrait ratio (HTMT), and cross-loadings; Fornell and Larcker, 1981; Henseler et al., 2015; Hair et al., 2022].

Because all constructs were measured with a single self-report questionnaire, common method bias was addressed procedurally, by assuring anonymity and stating that there were no right or wrong answers (Podsakoff et al., 2003), and statistically, using the full collinearity assessment approach (Kock, 2015). In this approach, all constructs are regressed on a randomly generated variable, and variance inflation factor (VIF) values above 3.3 indicate potential common method bias.

The structural model was assessed through collinearity (inner VIF), path coefficients, coefficients of determination (R^2^), and effect sizes (f^2^). Significance was tested with bootstrapping (5,000 subsamples, percentile confidence intervals, two-tailed tests, α = 0.05, fixed random seed). Model fit was assessed with the standardized root mean square residual (SRMR). Out-of-sample predictive power was assessed with PLSpredict (10-fold, 10 repetitions; Shmueli et al., 2019) and the cross-validated predictive ability test (CVPAT; Sharma et al., 2023). Indirect effects were tested as specific indirect effects using the same bootstrapping procedure. Because the model does not include direct paths parallel to the hypothesized indirect paths, indirect effects were evaluated without classifying mediation as full or partial. The moderating effect was estimated with the two-stage approach and probed with simple slopes at one standard deviation above and below the mean of AI literacy (Hair et al., 2022).

## 5 Results

### 5.1 Sample characteristics

Table 1 presents the characteristics of the 342 respondents. The sample was 54.1% male, and most respondents (68.1%) were aged 20–30 years. Three quarters (75.1%) reported using AI frequently or very frequently.

**TABLE 1:** Characteristics of respondents (*N* = 342).

Category	Frequency	Percentage (%)
Gender		
Male	185	54.1
Female	157	45.9
Age group		
20–25 years	142	41.5
26–30 years	91	26.6
31–35 years	63	18.4
Above 35 years	46	13.5
AI usage frequency		
Very frequent	158	46.2
Frequent	99	28.9
Occasional	61	17.8
Rare	24	7.0

### 5.2 Measurement model

Table 2 reports the measurement model results. All indicator loadings ranged from 0.861 to 0.921, exceeding the recommended threshold of 0.708. Cronbach’s α ranged from 0.851 to 0.904, ρA from 0.853 to 0.905, and composite reliability (ρC) from 0.910 to 0.940, indicating satisfactory internal consistency reliability. AVE values ranged from 0.771 to 0.839, exceeding the threshold of 0.50 and supporting convergent validity.

**TABLE 2:** Indicator loadings, reliability, and convergent validity.

Construct	Item	Loading	α	ρA	ρC	AVE
AI explainability (AE)	AE1/AE2/AE3	0.895/0.897/0.915	0.886	0.890	0.929	0.814
Algorithmic transparency (AT)	AT1/AT2/AT3	0.868/0.893/0.883	0.857	0.861	0.913	0.777
Perceived AI accuracy (PA)	PA1/PA2/PA3	0.885/0.881/0.902	0.868	0.868	0.919	0.792
Cognitive understanding (CU)	CU1/CU2/CU3	0.917/0.909/0.921	0.904	0.905	0.940	0.839
Cognitive trust (CT)	CT1/CT2/CT3	0.913/0.918/0.915	0.903	0.904	0.939	0.838
Trust calibration (TC)	TC1/TC2/TC3	0.900/0.886/0.872	0.863	0.865	0.916	0.785
Human reliance on AI (HR)	HR1/HR2/HR3	0.882/0.901/0.886	0.868	0.869	0.919	0.792
AI literacy (AL)	AL1/AL2/AL3	0.881/0.861/0.892	0.851	0.853	0.910	0.771

α, Cronbach’s alpha; ρA and ρC, composite reliability; AVE, average variance extracted. Item wording is reported in Supplementary Table 1.

Discriminant validity was supported by three criteria. First, the square root of each construct’s AVE exceeded its correlations with all other constructs (Table 3). Second, all HTMT values were below the conservative threshold of 0.85, ranging from 0.145 to 0.728, and the upper bounds of all 95% bootstrap confidence intervals were also below 0.85 (maximum = 0.791; Table 4 and Supplementary Table 4). Third, each item loaded more highly on its own construct than on any other construct, with a minimum difference of 0.30 between an item’s own loading and its highest cross-loading (Supplementary Table 3). Notably, although AI literacy and cognitive understanding are conceptually related, their correlation was moderate (*r* = 0.377; HTMT = 0.429), and the cognitive understanding items loaded much more strongly on their own construct (0.909–0.921) than on AI literacy (0.332–0.368), supporting the conceptual distinction drawn in section “2.4 Cognitive transparency and related constructs.”

**TABLE 3:** Discriminant validity: Fornell-Larcker criterion.

Construct	AE	AT	PA	CU	CT	TC	HR	AL
AE	**0.902**	–	–	–	–	–	–	–
AT	0.584	**0.881**	–	–	–	–	–	–
PA	0.545	0.549	**0.890**	–	–	–	–	–
CU	0.518	0.514	0.393	**0.916**	–	–	–	–
CT	0.527	0.485	0.645	0.582	**0.915**	–	–	–
TC	0.296	0.385	0.471	0.370	0.631	**0.886**	–	–
HR	0.221	0.293	0.317	0.241	0.422	0.600	**0.890**	–
AL	0.518	0.536	0.547	0.377	0.467	0.291	0.123	**0.878**

Diagonal values (bold) are the square roots of average variance extracted (AVE); off-diagonal values are latent variable correlations. AE, AI explainability; AT, algorithmic transparency; PA, perceived AI accuracy; CU, cognitive understanding; CT, cognitive trust; TC, trust calibration; HR, human reliance on AI; AL, AI literacy.

**TABLE 4:** Discriminant validity: heterotrait–monotrait ratio (HTMT).

Construct	AE	AT	PA	CU	CT	TC	HR
AT	0.671	–	–	–	–	–	–
PA	0.622	0.638	–	–	–	–	–
CU	0.577	0.582	0.444	–	–	–	–
CT	0.589	0.552	0.728	0.643	–	–	–
TC	0.339	0.450	0.544	0.420	0.713	–	–
HR	0.250	0.341	0.365	0.272	0.476	0.692	–
AL	0.596	0.627	0.637	0.429	0.531	0.339	0.145

All values are below 0.85. The upper bounds of all 95% percentile bootstrap confidence intervals (5,000 subsamples) were also below 0.85 (maximum = 0.791 for PA–CT). AE, AI explainability; AT, algorithmic transparency; PA, perceived AI accuracy; CU, cognitive understanding; CT, cognitive trust; TC, trust calibration; HR, human reliance on AI; AL, AI literacy.

### 5.3 Common method bias

In the full collinearity assessment, the VIF values of all constructs ranged from 1.61 to 2.82 (Table 5), below the threshold of 3.3 (Kock, 2015). Together with the procedural remedies described in section “4.4 Data analysis,” this suggests that common method bias is unlikely to account for the results. Nevertheless, because all constructs were self-reported by the same respondents at the same time, common method variance cannot be ruled out entirely (see section “6.4 Limitations and future research”).

**TABLE 5:** Collinearity assessment.

Construct	Full collinearity VIF	Structural path	Inner VIF
AI explainability	1.96	AE → CU	1.695
Algorithmic transparency	2.00	AT → CU	1.712
Perceived AI accuracy	2.21	AL → CU	1.555
Cognitive understanding	1.79	AE × AL → CU	1.055
Cognitive trust	2.82	CU → CT	1.183
Trust calibration	2.20	PA → CT	1.183
Human reliance on AI	1.61	CT → TC	1.000
AI literacy	1.73	TC → HR	1.000

Full collinearity variance inflation factors (VIFs) were obtained by regressing all eight constructs on a randomly generated variable (Kock, 2015); inner VIFs refer to the predictors in the structural model. Threshold = 3.3. AE, AI explainability; AT, algorithmic transparency; PA, perceived AI accuracy; CU, cognitive understanding; CT, cognitive trust; TC, trust calibration; HR, human reliance on AI; AL, AI literacy.

### 5.4 Structural model

Inner VIF values ranged from 1.000 to 1.712 (Table 5), indicating that collinearity among predictor constructs was not a concern. Table 6 reports the structural model results. AI explainability (β = 0.349, *p* < 0.001) and algorithmic transparency (β = 0.297, *p* < 0.001) were positively associated with cognitive understanding, supporting H1 and H2. Cognitive understanding (β = 0.388, *p* < 0.001) and perceived AI accuracy (β = 0.492, *p* < 0.001) were positively associated with cognitive trust, supporting H3 and H4; the association was stronger for perceived accuracy than for cognitive understanding. Cognitive trust was positively associated with trust calibration (β = 0.631, *p* < 0.001), and trust calibration was positively associated with human reliance on AI (β = 0.600, *p* < 0.001), supporting H5 and H6. The main effect of AI literacy on cognitive understanding was not significant (β = 0.078, *p* = 0.153).

**TABLE 6:** Structural model results (direct effects).

Hypothesis	Path	β	SD	*t*	*P*	95% CI	f^2^	Result
H1	AE → CU	0.349	0.056	6.26	<0.001	[0.241, 0.458]	0.116	Supported
H2	AT → CU	0.297	0.052	5.73	<0.001	[0.196, 0.399]	0.083	Supported
H3	CU → CT	0.388	0.041	9.51	<0.001	[0.309, 0.469]	0.279	Supported
H4	PA → CT	0.492	0.039	12.76	<0.001	[0.415, 0.564]	0.448	Supported
H5	CT → TC	0.631	0.032	19.82	<0.001	[0.564, 0.689]	0.660	Supported
H6	TC → HR	0.600	0.037	16.01	<0.001	[0.523, 0.668]	0.561	Supported
Main effect	AL → CU	0.078	0.054	1.43	0.153	[−0.027, 0.184]	0.006	Not significant

The AI literacy main effect was estimated as part of the moderation analysis and was not hypothesized. β, standardized path coefficient; SD, bootstrap standard deviation; CI, percentile bootstrap confidence interval (5,000 subsamples); AE, AI explainability; AT, algorithmic transparency; PA, perceived AI accuracy; CU, cognitive understanding; CT, cognitive trust; TC, trust calibration; HR, human reliance on AI; AL, AI literacy.

Effect sizes (f^2^) were small for AI explainability (0.116) and algorithmic transparency (0.083) on cognitive understanding, medium for cognitive understanding on cognitive trust (0.279), and large for perceived accuracy on cognitive trust (0.448), cognitive trust on trust calibration (0.660), and trust calibration on human reliance (0.561).

The model explained 38.1% of the variance in cognitive understanding, 54.3% in cognitive trust, 39.8% in trust calibration, and 36.0% in human reliance on AI (Table 7), indicating moderate explanatory power. The SRMR of the estimated model was 0.048, below the recommended threshold of 0.08.

**TABLE 7:** Coefficients of determination.

Endogenous construct	R^2^	Adjusted R^2^	95% CI for R^2^
Cognitive understanding	0.381	0.374	[0.310, 0.464]
Cognitive trust	0.543	0.540	[0.476, 0.610]
Trust calibration	0.398	0.396	[0.318, 0.475]
Human reliance on AI	0.360	0.358	[0.274, 0.447]

Table 8 reports the PLSpredict results. All Q^2^predict values for the indicators of the endogenous constructs exceeded zero (range = 0.074–0.395), indicating that the model outperforms the naïve mean benchmark. The PLS-SEM model yielded lower root mean square error (RMSE) values than the linear model benchmark for 11 of the 12 indicators, indicating medium predictive power (Shmueli et al., 2019). Predictive relevance was highest for cognitive trust (construct-level Q^2^predict = 0.467) and cognitive understanding (0.357), moderate for trust calibration (0.236), and lowest for human reliance on AI (0.102). CVPAT showed that the model’s predictive performance was significantly better than the indicator-average benchmark for all endogenous constructs (overall average loss difference = −0.174, *p* < 0.001) and better than the linear model benchmark overall (average loss difference = −0.026, *p* < 0.001), although the difference relative to the linear model was not significant for human reliance (*p* = 0.348).

**TABLE 8:** Out-of-sample predictive power (PLSpredict).

Indicator	Q^2^predict	PLS-SEM RMSE	LM RMSE	PLS-SEM MAE	LM MAE
CU1	0.300	0.696	0.717	0.561	0.577
CU2	0.294	0.726	0.763	0.583	0.616
CU3	0.301	0.697	0.723	0.570	0.582
CT1	0.386	0.651	0.672	0.519	0.535
CT2	0.395	0.671	0.689	0.536	0.549
CT3	0.389	0.666	0.681	0.540	0.549
TC1	0.167	0.825	0.846	0.671	0.680
TC2	0.224	0.758	0.771	0.617	0.619
TC3	0.163	0.779	0.794	0.634	0.634
HR1	0.074	0.857	0.870	0.706	0.701
HR2	0.086	0.841	0.841	0.694	0.672
HR3	0.082	0.820	0.833	0.674	0.670

PLSpredict settings: 10 folds, 10 repetitions. LM, linear model benchmark; RMSE, root mean square error; MAE, mean absolute error. For HR2, the partial least squares structural equation modeling (PLS-SEM) RMSE (0.8414) was marginally higher than the LM RMSE (0.8410).

### 5.5 Indirect effects

Table 9 reports the specific indirect effects. The indirect effect of AI explainability on cognitive trust through cognitive understanding was significant (β = 0.135, 95% CI [0.087, 0.191]), supporting H8. The indirect effect of cognitive understanding on trust calibration through cognitive trust was significant (β = 0.245, 95% CI [0.190, 0.301]), supporting H9. The indirect effect of cognitive trust on human reliance through trust calibration was significant (β = 0.378, 95% CI [0.315, 0.441]), supporting H10. The serial indirect effect spanning the full sequence from AI explainability to human reliance was also significant, although small (β = 0.051, 95% CI [0.032, 0.075]). In addition, algorithmic transparency had a significant, non-hypothesized indirect association with cognitive trust through cognitive understanding (β = 0.115, 95% CI [0.071, 0.163]). Because the data are cross-sectional, these results indicate that the associations are statistically consistent with the proposed sequence; they do not establish causal mediation.

**TABLE 9:** Specific indirect effects.

Hypothesis	Indirect path	β	SD	*t*	*P*	95% CI	Result
H8	AE → CU → CT	0.135	0.027	5.08	<0.001	[0.087, 0.191]	Supported
H9	CU → CT → TC	0.245	0.028	8.67	<0.001	[0.190, 0.301]	Supported
H10	CT → TC → HR	0.378	0.032	11.73	<0.001	[0.315, 0.441]	Supported
Additional	AE → CU → CT → TC → HR	0.051	0.011	4.70	<0.001	[0.032, 0.075]	Significant
Additional	AT → CU → CT	0.115	0.024	4.85	<0.001	[0.071, 0.163]	Significant

Percentile bootstrap confidence intervals (5,000 subsamples). Rows labeled Additional were not hypothesized and are reported for completeness: the full serial indirect effect and the indirect effect of algorithmic transparency on cognitive trust. AE, AI explainability; AT, algorithmic transparency; PA, perceived AI accuracy; CU, cognitive understanding; CT, cognitive trust; TC, trust calibration; HR, human reliance on AI; AL, AI literacy.

### 5.6 Moderation analysis

The interaction between AI explainability and AI literacy was positive and significant (β = 0.192, *t* = 5.06, *p* < 0.001, 95% CI [0.119, 0.268], f^2^ = 0.071), supporting H7 (Table 10). Simple slope analysis showed that the association between AI explainability and cognitive understanding was stronger at high AI literacy (+1 SD: β = 0.541, *p* < 0.001) than at the mean (β = 0.349, *p* < 0.001) and at low AI literacy (−1 SD: β = 0.156, *p* = 0.014), although it remained significant at all three levels (Figure 2). Consistent with this, the indirect association of AI explainability with cognitive trust through cognitive understanding was also moderated by AI literacy (index of moderated mediation = 0.075, 95% CI [0.044, 0.111]), with conditional indirect effects of 0.061 at low, 0.135 at mean, and 0.210 at high AI literacy.

**TABLE 10:** Moderating effect of artificial intelligence (AI) literacy.

Effect	β	SD	*t*	*P*	95% CI
AE × AL → CU (H7)	0.192	0.038	5.06	<0.001	[0.119, 0.268]
Simple slope AE → CU at AL −1 SD	0.156	0.063	2.47	0.014	[0.031, 0.283]
Simple slope AE → CU at AL mean	0.349	0.056	6.26	<0.001	[0.241, 0.458]
Simple slope AE → CU at AL +1 SD	0.541	0.071	7.60	<0.001	[0.404, 0.680]

The interaction term was estimated with the two-stage approach; f^2^ of the interaction term = 0.071. AE, AI explainability; AT, algorithmic transparency; PA, perceived AI accuracy; CU, cognitive understanding; CT, cognitive trust; TC, trust calibration; HR, human reliance on AI; AL, AI literacy.

**FIGURE 2:**
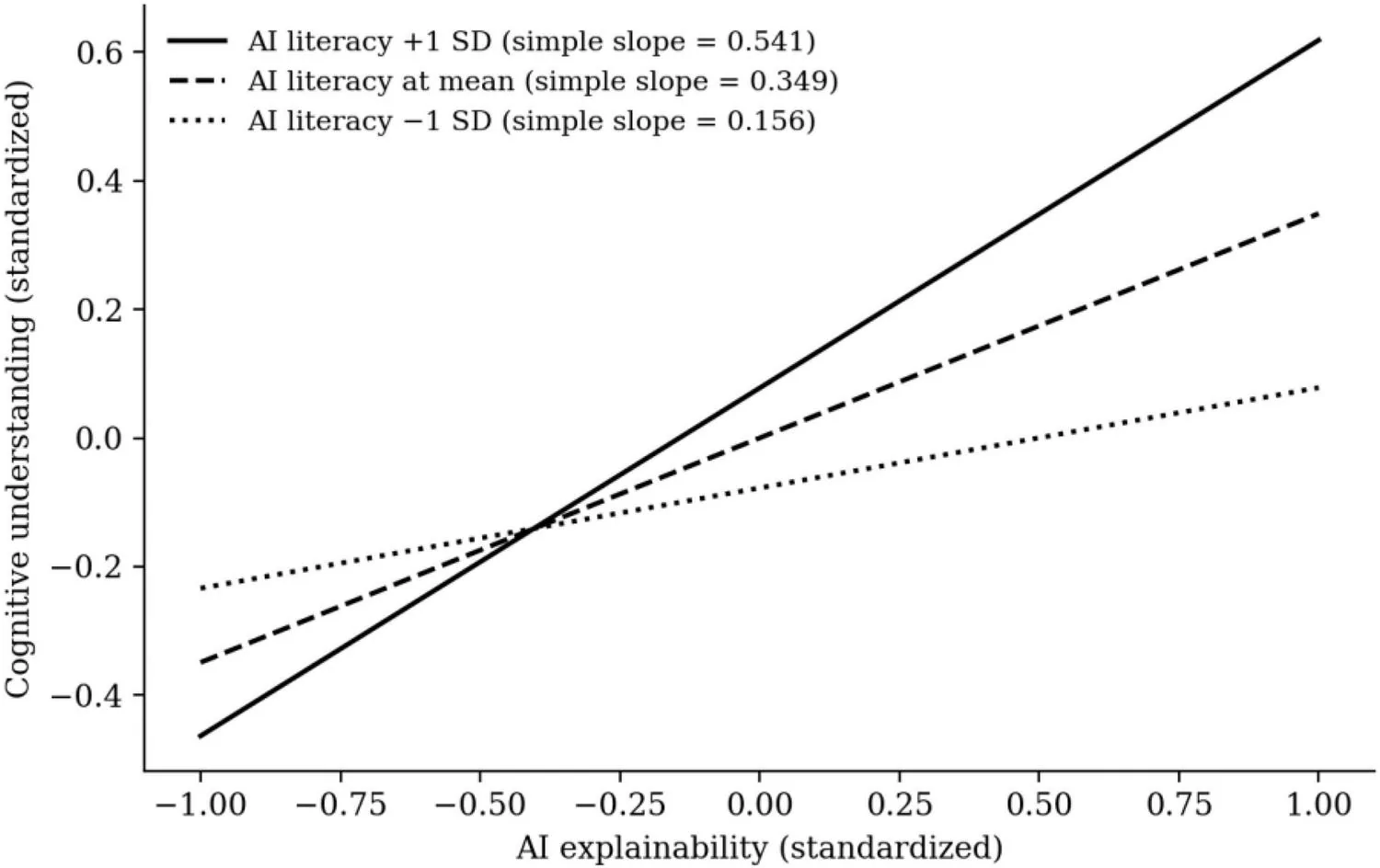
Simple slopes of AI explainability on cognitive understanding at low (−1 SD), mean, and high (+1 SD) levels of AI literacy.

### 5.7 Summary of hypothesis tests

Table 11 summarizes the results of the hypothesis tests. All seven direct and moderating hypotheses (H1-H7) and all three indirect-effect hypotheses (H8-H10) were supported. As noted in the table, these results concern associations among self-reported perceptions in cross-sectional data.

**TABLE 11:** Summary of hypothesis tests.

Hypothesis	Proposed association	Result
H1	AI explainability → cognitive understanding (+)	Supported
H2	Algorithmic transparency → cognitive understanding (+)	Supported
H3	Cognitive understanding → cognitive trust (+)	Supported
H4	Perceived AI accuracy → cognitive trust (+)	Supported
H5	Cognitive trust → trust calibration (+)	Supported
H6	Trust calibration → human reliance on AI (+)	Supported
H7	AI literacy strengthens AI explainability → cognitive understanding	Supported
H8	AI explainability → cognitive understanding → cognitive trust	Supported
H9	Cognitive understanding → cognitive trust → trust calibration	Supported
H10	Cognitive trust → trust calibration → human reliance	Supported

All hypotheses concern associations among self-reported perceptions in cross-sectional data.

## 6 Discussion

### 6.1 Discussion of main findings

This study tested a model linking users’ perceptions of explainable AI to their human reliance on AI through cognitive understanding, cognitive trust, and trust calibration, with AI literacy as a moderator. All hypothesized associations were supported, and the pattern of results is consistent with the proposed sequence. Because all constructs were self-reported and measured at one point in time, the findings describe associations among users’ perceptions rather than causal effects.

First, participants who perceived their chosen AI system as more explainable and transparent reported understanding it better. This is consistent with the view that explanations support the formation of mental models (Bansal et al., 2019; Kelly et al., 2023; Shin, 2021). The effect sizes of these associations were small, however, and the two perceptions together with AI literacy explained 38% of the variance in cognitive understanding. This suggests that users’ sense of understanding an AI system depends on much more than whether it appears to explain itself.

Second, both cognitive understanding and perceived accuracy were associated with cognitive trust, but perceived accuracy showed the stronger association (β = 0.492 versus 0.388; f^2^ = 0.448 versus 0.279). This is consistent with trust theory’s emphasis on perceived competence (Mayer et al., 1995) and with research identifying perceived performance as a central determinant of trust in automation (Hoff and Bashir, 2015; Glikson and Woolley, 2020). It suggests that, in users’ own evaluations, whether a system appears to get things right weighs more heavily than whether they feel they understand how it works.

Third, cognitive trust was strongly associated with trust calibration, which in turn was strongly associated with human reliance. Users who trusted their system more were more likely to feel that their trust matched its performance and that they could judge when to trust it, and these users reported relying more on AI. These findings should be interpreted with the distinction drawn in section “2.2 Trust in automation and trust calibration” in mind. The measure captures users’ belief that their trust is well calibrated, not whether it actually is. The strong association between cognitive trust and perceived calibration is also compatible with an alternative interpretation: users who trust a system may simply feel confident in their trust, including when that trust is excessive. Distinguishing these interpretations requires independent measures of system performance. This concern is supported by recent evidence that users often overestimate the accuracy of AI outputs, particularly when explanations are provided (Steyvers et al., 2025), and that people’s confidence judgments about AI can be poorly aligned with its actual accuracy (Steyvers and Peters, 2026).

Additional analyses, which were not hypothesized in advance, provide further context. The full serial indirect effect from AI explainability to human reliance on AI through cognitive understanding, cognitive trust, and trust calibration was significant (β = 0.051, 95% CI [0.032, 0.075]), supporting the proposed sequence as a whole rather than only its individual links. Algorithmic transparency was also indirectly associated with cognitive trust through cognitive understanding (β = 0.115, 95% CI [0.071, 0.163]), suggesting that both explainability and transparency relate to trust through users’ understanding. Because these effects were exploratory, they should be confirmed in future research.

Fourth, AI literacy strengthened the association between AI explainability and cognitive understanding: this association was more than three times as strong among users with high AI literacy as among those with low AI literacy. AI literacy itself had no significant direct association with cognitive understanding once AI explainability was taken into account. This pattern is consistent with the argument that general AI knowledge helps users make sense of a particular system’s explanations (Long and Magerko, 2020), rather than directly producing system-specific understanding.

Finally, the model’s predictive power was medium overall but weak for human reliance (Q^2^predict = 0.102). Reliance on AI is therefore only partly accounted for by the understanding- and trust-related judgments examined here, and other factors, such as task demands, habit, perceived risk, or organizational norms, are likely to play a substantial role.

These findings extend prior work in a specific way. Technology acceptance frameworks such as TAM and UTAUT explain use through beliefs about usefulness and ease of use (Davis, 1989; Venkatesh et al., 2003), and many XAI studies examine explainability as a direct antecedent of trust or acceptance (Shin, 2021; Dwivedi et al., 2023). By contrast, the present model decomposes the link between perceptions of explainable AI and reliance into a sequence of understanding-, trust-, and calibration-related judgments and tests these jointly. The results show that, within this framework, perceptions of explainability are only indirectly and modestly related to reliance (serial indirect effect β = 0.051), whereas perceived accuracy and trust-related judgments are more strongly related to it.

### 6.2 Theoretical implications

First, the study contributes to XAI research by showing that the associations between users’ perceptions of explainability and their reliance on AI are consistent with a sequence running through cognitive understanding, cognitive trust, and trust calibration. This supports theoretical arguments that the relationship between explainability and reliance is psychologically mediated rather than direct, although longitudinal and experimental research is needed to establish the ordering of these processes.

Second, the study contributes to trust theory by showing that cognitive understanding and perceived accuracy are distinct and complementary correlates of cognitive trust in AI, with perceived accuracy showing the stronger association. Models of trust in AI that focus exclusively on explainability may therefore underestimate the role of perceived performance.

Third, the study contributes to trust in automation research by distinguishing perceived from actual trust calibration. The strong associations involving perceived calibration show that users’ subjective sense that their trust is appropriate is closely tied to both their trust and their reliance. Whether this subjective sense corresponds to actual calibration is an open question with important implications, because a confident but inaccurate sense of calibration could sustain overreliance.

Fourth, the study contributes to research on AI literacy by showing that general AI knowledge conditions the association between AI explainability and system-specific understanding. This supports conceptualizing AI literacy as a boundary condition of explainable AI rather than as an equivalent of system-specific understanding.

### 6.3 Practical implications

The findings suggest several practical implications, which should be considered in light of the study’s correlational design. First, because perceived accuracy was the strongest correlate of trust, AI developers should give users accessible information about a system’s performance, such as accuracy rates, confidence scores, and known failure conditions, alongside explanations of individual outputs. Second, because AI explainability was associated with understanding more strongly among users with higher AI literacy, explanation features are likely to be most useful when combined with AI literacy support. Organizations deploying AI decision-support tools in domains such as healthcare, education, and finance could pair user-facing explanation modules with brief training on how AI systems generate outputs and where they tend to fail. Third, because users’ sense that their trust is well-calibrated may not reflect actual calibration, organizations should not rely on users’ self-assessments alone. Instead, they could provide feedback on the system’s performance in users’ own decisions, for example by periodically showing users cases in which the AI was wrong, so that users can check their trust against evidence (Mehrotra et al., 2024). Finally, policymakers promoting transparency requirements for AI should recognize that explanations are associated with understanding only modestly on their own, and that transparency measures are likely to be more effective when accompanied by AI literacy initiatives.

### 6.4 Limitations and future research

This study has several limitations. First, the cross-sectional design does not allow causal or temporal ordering to be established. Although the model specifies a theoretically derived sequence, alternative orderings are plausible: for example, users who trust a system may consequently feel that they understand it, and experience of relying on a system may shape trust rather than result from it. The significant indirect effects show only that the data are consistent with the proposed sequence. Similarly, the theoretical view that trust develops dynamically over repeated interactions could not be examined. Longitudinal designs that measure these constructs repeatedly over the course of interaction with a system, and experimental designs that manipulate explanation features, are needed to establish direction and dynamics.

Second, all constructs were measured through self-report. Self-reported cognitive understanding does not establish demonstrated comprehension, self-assessed trust calibration does not establish that trust corresponds to a system’s actual capabilities, and self-reported human reliance on AI does not establish that reliance was appropriate. The findings should therefore not be interpreted as evidence that explainability produces appropriately calibrated reliance. Future research should combine self-report measures with objective indicators, such as comprehension tests, comparisons of trust with system accuracy, and behavioral measures of reliance, such as the rate at which users follow correct and incorrect AI recommendations (Buçinca et al., 2021; Lai et al., 2023; Klingbeil et al., 2024).

Third, one trust calibration item (“I rely on the system appropriately”) refers to reliance. Although discriminant validity between trust calibration and human reliance was supported (HTMT = 0.692), this wording may have contributed to the strength of their association. Future measures of perceived calibration should avoid reference to reliance.

Fourth, participants evaluated a self-selected AI system, and the type of system was not recorded. Because these systems likely differed in their explanation features, transparency, and accuracy, the findings reflect relationships among users’ perceptions across a heterogeneous range of AI systems rather than responses to any specific system. This enhances ecological validity but limits conclusions about particular system types. Future research should record or standardize the reference system.

Fifth, the measurement items were developed for this study based on the relevant literature, and no formal pilot test or expert panel review was conducted before data collection. Although the measurement model showed satisfactory reliability and convergent and discriminant validity, this evidence derives from the present sample only, and three-item measures may not capture the full breadth of each construct. In particular, the AI literacy measure captures self-reported knowledge and familiarity and includes an item on confidence in using AI tools, which overlaps with self-efficacy; it does not assess the broader competencies described in AI literacy frameworks (Long and Magerko, 2020; UNESCO, 2024). Future research should establish content validity through expert review, pilot-test the items, validate the measures in independent samples, and compare them with established instruments where available.

Sixth, the questionnaire was administered in English, which may have affected comprehension for some participants whose first language is Korean. In addition, the convenience sample of university students and early-career professionals in South Korea limits generalizability to other populations, age groups, and cultural contexts.

Finally, although the full collinearity assessment did not indicate common method bias, all data came from a single self-report survey, and common method variance may have inflated the associations among constructs. Future studies could collect predictor and outcome measures at different times or from different sources.

## 7 Conclusion

This study examined how users’ perceptions of explainable AI are related to their human reliance on AI. In a sample of 342 AI users in South Korea, AI explainability and algorithmic transparency were positively associated with cognitive understanding; cognitive understanding and, more strongly, perceived accuracy were associated with cognitive trust; cognitive trust was associated with trust calibration; and trust calibration was associated with human reliance. AI literacy strengthened the association between AI explainability and cognitive understanding. The pattern of results is consistent with a sequence in which perceptions of explainability relate to reliance through understanding- and trust-related judgments, although the cross-sectional, self-report design does not allow causal conclusions or conclusions about whether reliance was objectively appropriate.

For practice, the findings suggest that AI systems should integrate user-facing explanation modules with accessible information about system accuracy and limitations, and that organizations should accompany AI deployment with AI literacy training and feedback that allows users to check their trust against the system’s actual performance. For research, the central next step is to test these relationships with experimental and longitudinal designs that include objective measures of comprehension, calibration, and appropriate reliance.

## Data Availability

The raw data supporting the conclusions of this article will be made available by the author, without undue reservation.
