# Supplementary material for: Cognitive transparency and trust calibration in explainable AI systems: a psychological model of human-AI decision reliance
Source: Front Psychol. 2026 Oct 14;17:1924913. doi: 10.3389/fpsyg.2026.1924913 (PMC13713401; doi:10.3389/fpsyg.2026.1924913)
Supplement: Supplementary file 1 [file Data_Sheet_1.pdf]

TYPE Supplementary Material

Supplementary Material

Usman Rehman*
Department of Global Venture Technology Management, Hoseo University, Seoul, Republic of Korea
*CORRESPONDENCE Usman Rehman, usmanrehman001@hoseo.edu

Supplementary material for: *Cognitive Transparency and Trust Calibration in Explainable AI Systems: A Psychological Model of Human-AI Decision Reliance*

S1 Survey instructions, response format, and item development

Survey instructions (as administered, in English). "Please think about an AI-based system or application that you have personally used and are familiar with. This may include generative AI platforms, AI-powered recommendation systems, intelligent assistants, predictive analytics applications, or other AI-supported decision-making systems. For all questions that follow, please keep the same AI system in mind and answer based on your actual experience with that system. Please indicate the response that best reflects your perceptions, experiences, and interactions with the AI system. There are no right or wrong answers."

Response format and scoring. All items were rated on a five-point Likert scale (1 = strongly disagree, 2 = disagree, 3 = neutral, 4 = agree, 5 = strongly agree). No items were reverse-coded. Item responses were entered as reflective indicators in SmartPLS 4, which estimated construct scores as weighted composites of their indicators.

Item development. All items were developed for this study, informed by the literature listed in Supplementary Table S2. The cited works provide the conceptual basis for each construct; they are not sources of previously published scales. Items refer to the participant's self-selected AI system, except for the AI literacy items, which refer to AI technologies in general. No formal pilot test or expert panel review was conducted before data collection. The questionnaire was administered in English.

TABLE S1 Measurement items and descriptive statistics (N = 342).

Construct	Code	Item	M	SD
AI explainability	AE1	The AI system provides clear explanations for its decisions	3.85	0.87
	AE2	I understand why the AI system generated specific outputs	3.87	0.82
	AE3	The system explains its decisions in an understandable way	3.81	0.85
Algorithmic transparency	AT1	The AI system is transparent in how it processes information	3.68	0.92
	AT2	I can see how the system reaches its decisions	3.73	0.83

Construct	Code	Item	M	SD
	AT3	The decision-making process of the AI is visible to me	3.68	0.87
Perceived AI accuracy	PA1	The AI system provides accurate results	3.76	0.83
	PA2	I trust the correctness of the system's outputs	3.78	0.83
	PA3	The system performs reliably	3.75	0.84
Cognitive understanding	CU1	I understand how the AI system works	3.66	0.83
	CU2	I can interpret the system's outputs	3.65	0.86
	CU3	I have a clear understanding of its decision-making process	3.63	0.83
Cognitive trust	CT1	I trust the AI system	3.59	0.83
	CT2	The system is reliable	3.64	0.86
	CT3	I have confidence in its performance	3.55	0.85
Trust calibration	TC1	My level of trust in the AI system matches its actual performance	3.57	0.90
	TC2	I rely on the system appropriately	3.57	0.86
	TC3	I can judge when to trust the system	3.59	0.85
Human reliance on AI	HR1	I rely on AI for decision-making	3.49	0.89
	HR2	I use AI recommendations in my decisions	3.47	0.88
	HR3	I depend on AI support	3.50	0.85
AI literacy	AL1	I understand AI technologies	3.73	0.87
	AL2	I am familiar with how AI systems work	3.68	0.86
	AL3	I feel confident using AI tools	3.67	0.88

Note. Item codes correspond to the variable names in the raw data file. M = mean; SD = standard deviation.

TABLE S2 Conceptual basis of the measures.

Construct	Conceptual basis for item development
AI explainability	Explainable AI literature (Ribeiro et al., 2016; Adadi and Berrada, 2018; Miller, 2019)
Algorithmic transparency	Transparency and explainability literature (Miller, 2019; Shin, 2021)
Perceived AI accuracy	Trust in automation literature on perceived reliability and performance (Hoff and Bashir, 2015)
Cognitive understanding	Explainability and mental model literature (Shin, 2021; Kelly et al., 2023)
Cognitive trust	Trust theory (Mayer et al., 1995; McKnight et al., 2002)
Trust calibration	Trust calibration theory (Lee and See, 2004)
Human reliance on AI	Automation reliance literature (Hoff and Bashir, 2015)
AI literacy	AI literacy literature (Long and Magerko, 2020)

S2 Additional discriminant validity results

TABLE S3 Indicator loadings and cross-loadings.

Item	AE	AT	PA	CU	CT	TC	HR	AL
AE1	.895	.505	.463	.445	.453	.241	.177	.465
AE2	.897	.529	.517	.451	.480	.279	.193	.488

Item	AE	AT	PA	CU	CT	TC	HR	AL
AE3	.915	.546	.497	.502	.493	.280	.224	.452
AT1	.546	.868	.503	.421	.423	.368	.291	.467
AT2	.512	.893	.466	.485	.416	.320	.278	.485
AT3	.490	.883	.485	.449	.446	.335	.207	.465
PA1	.478	.495	.885	.355	.569	.386	.257	.485
PA2	.480	.458	.881	.323	.582	.446	.292	.483
PA3	.498	.513	.902	.372	.570	.425	.297	.492
CU1	.470	.497	.387	.917	.546	.351	.214	.368
CU2	.463	.452	.337	.909	.509	.351	.226	.332
CU3	.490	.463	.355	.921	.542	.316	.222	.336
CT1	.498	.436	.582	.545	.913	.549	.362	.399
CT2	.462	.441	.602	.533	.918	.602	.398	.439
CT3	.489	.456	.586	.519	.915	.580	.397	.443
TC1	.233	.307	.406	.318	.584	.900	.560	.227
TC2	.307	.399	.459	.348	.578	.886	.506	.306
TC3	.248	.320	.386	.320	.512	.872	.526	.242
HR1	.133	.269	.270	.215	.352	.520	.882	.140
HR2	.201	.282	.295	.203	.372	.540	.901	.106
HR3	.253	.233	.280	.225	.402	.539	.886	.084
AL1	.424	.453	.481	.319	.405	.261	.127	.881
AL2	.444	.487	.489	.327	.388	.225	.103	.861
AL3	.494	.472	.472	.347	.435	.279	.096	.892

Note. Each item loads more highly on its own construct than on any other construct; the minimum difference between an item's own loading and its highest cross-loading is 0.30. AE = AI explainability; AT = algorithmic transparency; PA = perceived AI accuracy; CU = cognitive understanding; CT = cognitive trust; TC = trust calibration; HR = human reliance on AI; AL = AI literacy.

TABLE S4 HTMT values with 95% bootstrap confidence intervals.

Pair	HTMT	2.5%	97.5%
AE–AT	.671	.592	.743
AE–PA	.622	.540	.696
AE–CU	.577	.491	.654
AE–CT	.589	.507	.664
AE–TC	.339	.231	.442
AE–HR	.250	.141	.360
AE–AL	.596	.508	.678
AT–PA	.638	.552	.716
AT–CU	.582	.498	.662
AT–CT	.552	.465	.630
AT–TC	.450	.352	.543
AT–HR	.341	.225	.451
AT–AL	.627	.543	.703
PA–CU	.444	.338	.545
PA–CT	.728	.655	.791
PA–TC	.544	.442	.632

Pair	HTMT	2.5%	97.5%
PA–HR	.365	.259	.464
PA–AL	.637	.553	.716
CU–CT	.643	.568	.713
CU–TC	.420	.321	.514
CU–HR	.272	.170	.373
CU–AL	.429	.327	.527
CT–TC	.713	.641	.775
CT–HR	.476	.374	.568
CT–AL	.531	.434	.620
TC–HR	.692	.607	.765
TC–AL	.339	.223	.451
HR–AL	.145	.055	.263

Note. Percentile bootstrap confidence intervals based on 5,000 subsamples. All upper bounds are below 0.85. Abbreviations as in Table S3.

S3 Supplementary references

Adadi, A., and Berrada, M. (2018). Peeking inside the black-box: a survey on explainable artificial intelligence (XAI). *IEEE Access* 6, 52138–52160. doi: 10.1109/ACCESS.2018.2870052

Hoff, K. A., and Bashir, M. (2015). Trust in automation: integrating empirical evidence on factors that influence trust. *Hum. Factors* 57, 407–434. doi: 10.1177/0018720814547570

Kelly, S., Kaye, S. A., and Oviedo-Trespalacios, O. (2023). What factors contribute to the acceptance of artificial intelligence? A systematic review. *Telemat. Inform.* 77, 101925. doi: 10.1016/j.tele.2022.101925

Lee, J. D., and See, K. A. (2004). Trust in automation: designing for appropriate reliance. *Hum. Factors* 46, 50–80. doi: 10.1518/hfes.46.1.50_30392

Long, D., and Magerko, B. (2020). "What is AI literacy? Competencies and design considerations," in *Proceedings of the 2020 CHI Conference on Human Factors in Computing Systems* (New York, NY: ACM), 1–16. doi: 10.1145/3313831.3376727

Mayer, R. C., Davis, J. H., and Schoorman, F. D. (1995). An integrative model of organizational trust. *Acad. Manag. Rev.* 20, 709–734. doi: 10.5465/amr.1995.9508080335

McKnight, D. H., Choudhury, V., and Kacmar, C. (2002). Developing and validating trust measures for e-commerce: an integrative typology. *Inf. Syst. Res.* 13, 334–359. doi: 10.1287/isre.13.3.334.81

Miller, T. (2019). Explanation in artificial intelligence: insights from the social sciences. *Artif. Intell.* 267, 1–38. doi: 10.1016/j.artint.2018.07.007

Ribeiro, M. T., Singh, S., and Guestrin, C. (2016). "'Why should I trust you?' Explaining the predictions of any classifier," in *Proceedings of the 22nd ACM SIGKDD International Conference on Knowledge Discovery and Data Mining* (New York, NY: ACM), 1135–1144. doi: 10.1145/2939672.2939778

Shin, D. (2021). The effects of explainability and causability on perception, trust, and acceptance: implications for explainable AI. *Int. J. Hum. Comput. Stud.* 146, 102551. doi: 10.1016/j.ijhcs.2020.102551
